# Application of Magnetic Nanoparticles for Rapid Detection and In Situ Diagnosis in Clinical Oncology

**DOI:** 10.3390/cancers14020364

**Published:** 2022-01-12

**Authors:** Tatsuya Onishi, Kisyo Mihara, Sachiko Matsuda, Satoshi Sakamoto, Akihiro Kuwahata, Masaki Sekino, Moriaki Kusakabe, Hiroshi Handa, Yuko Kitagawa

**Affiliations:** 1 Department of Breast Surgery, National Cancer Center Hospital East, 6-5-1, Kashiwanoha, Kashiwa 277-8577, Chiba, Japan; t.onishi77@gmail.com; 2 Department of Surgery, Kawasaki Municipal Kawasaki Hospital, Kawasaki-ku, Kawasaki 210-0013, Kanagawa, Japan; kisyomihara@gmail.com; 3 Department of Surgery, School of Medicine, Keio University, 35 Shinanomachi, Shinjuku-ku, Tokyo 160-8582, Japan; kitagawa@a3.keio.jp; 4 School of Life Science and Technology, Tokyo Institute of Technology, 4259 Nagatsuta-cho, Midori-ku, Yokohama 226-8501, Kanagawa, Japan; ssakamoto@bio.titech.ac.jp; 5 Graduate School of Engineering, Tohoku University, 6-6-05 Aoba, Aramaki-aza, Aoba-ku, Sendai 980-8579, Miyagi, Japan; akihiro.kuwahata.b1@tohoku.ac.jp; 6 Graduate School of Engineering, The University of Tokyo, 7-3-1 Hongo, Bunkyo-ku, Tokyo 113-8656, Japan; sekino@g.ecc.u-tokyo.ac.jp; 7 Graduate School of Agricultural and Life Sciences, Research Center for Food Safety, The University of Tokyo, 1-1-1 Yayoi, Bunkyo-ku, Tokyo 113-8657, Japan; kusabmrl@gmail.com; 8 Matrix Cell Research Institute Inc., 1-35-3 Kamikashiwada, Ushiku 300-1232, Ibaraki, Japan; 9 Department of Nanoparticle Translational Research, Tokyo Medical University, 6-1-1 Shinjuku, Shinjuku-ku, Tokyo 160-8402, Japan; hhanda@tokyo-med.ac.jp

**Keywords:** magnetic nanoparticles, in situ diagnosis, rapid detection, extracellular vesical quantification, presurgical screening, pathological diagnosis, sentinel node mapping

## Abstract

**Simple Summary:**

Screening, monitoring, and diagnostic methods in oncology are a critical part of treatment. The currently used clinical methods have limitations, most notably the time, cost, and special facilities required for radioisotope-based techniques. The use of magnetic nanoparticles is an alternative approach that offers faster analyses with safer materials over a wide range of oncological applications, such as the detection of cancer biomarkers and immunostaining. Furthermore, magnetic nanoparticles, such as superparamagnetic iron oxide nanoparticles, can detect sentinel lymph nodes for breast cancer in a clinical setting, as well as those for gallbladder cancer in animal models within a timeframe that would enable them to be used during surgery with a magnetic probe.

**Abstract:**

Screening, monitoring, and diagnosis are critical in oncology treatment. However, there are limitations with the current clinical methods, notably the time, cost, and special facilities required for radioisotope-based methods. An alternative approach, which uses magnetic beads, offers faster analyses with safer materials over a wide range of oncological applications. Magnetic beads have been used to detect extracellular vesicles (EVs) in the serum of pancreatic cancer patients with statistically different EV levels in preoperative, postoperative, and negative control samples. By incorporating fluorescence, magnetic beads have been used to quantitatively measure prostate-specific antigen (PSA), a prostate cancer biomarker, which is sensitive enough even at levels found in healthy patients. Immunostaining has also been incorporated with magnetic beads and compared with conventional immunohistochemical methods to detect lesions; the results suggest that immunostained magnetic beads could be used for pathological diagnosis during surgery. Furthermore, magnetic nanoparticles, such as superparamagnetic iron oxide nanoparticles (SPIONs), can detect sentinel lymph nodes in breast cancer in a clinical setting, as well as those in gallbladder cancer in animal models, in a surgery-applicable timeframe. Ultimately, recent research into the applications of magnetic beads in oncology suggests that the screening, monitoring, and diagnosis of cancers could be improved and made more accessible through the adoption of this technology.

## 1. Introduction

Magnetic nanoparticles (MNPs) have recently been applied to life sciences as well as clinical settings. MNPs comprise aggregates of iron oxide (FeO, Fe_2_O_3_, and Fe_3_O_4_) or ferrite particles (which contain iron oxide as the main component) in the nanometer order, which are dispersed or embedded in polymers, such as polysaccharide, polystyrene, silica, and agarose [1]. Their application to life science research stems from the ability to separate, guide, and detect MNPs using magnetic fields. Additionally, MNPs can be processed to furnish their surface with a variety of functions. Recognition sites, such as functional groups and biomolecules, are immobilized on the surface of the beads and are used to recognize targets for separation or detection [1]. The physical size and magnetization strength of the beads are roughly proportional to the number of iron oxide particles in the polymer. Protein purification and cell separation applications require strong magnetic particles, whereby micro-sized magnetic particles are used with a magnetic field [2]. For stem cell differentiation experiments and gene transfer applications, small magnetic particles (<100 nm) are generally used [3]. Furthermore, some nanosized magnetic particles, such as superparamagnetic iron oxide nanoparticles (SPIONs), are biocompatible and can be used internally in magnetic resonance imaging (MRI) contrast media for the liver [4].

**Figure 1:**
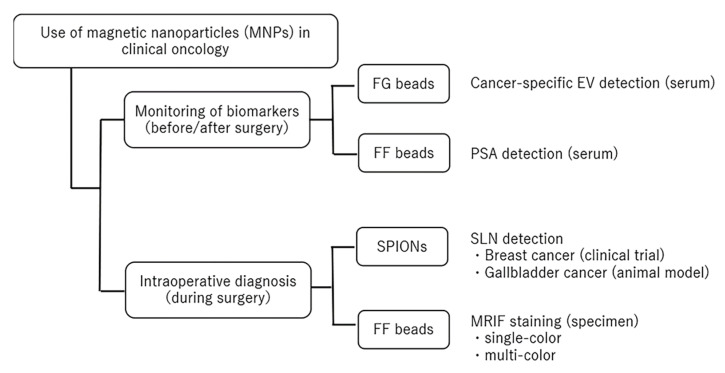
The concept of this review. The usage of magnetic nanoparticles (MNPs) was divided into two objectives: monitoring of biomarkers (before/after surgery) and intraoperative diagnosis (during surgery). The types of MNPs and examples of their use are indicated. FG, ferrite and glycidyl methacrylate; FF, fluorescent FG; SPIONs, superparamagnetic iron oxide nanoparticles; EVs, extracellular vesicles; PSA, prostate-specific antigen; SLN, sentinel lymph node; MRIF, magnetically promoted rapid immunofluorescence.

We focused on the applications of MNPs in oncology from a surgeon’s perspective when monitoring biomarkers before and after surgery, and for intraoperative diagnosis during surgery (Figure 1). In this review, we provide an overview of the application of MNPs in oncology.

## 2. Monitoring Biomarkers before and after Surgery

In oncological clinical settings, early detection and accurate diagnosis are important for cancer treatment, both before and after surgery. Enzyme-linked immuno-sorbent assay (ELISA) [5,6,7], which uses antigen–antibody reactions as its detection mechanism, is widely used to detect cancer biomarkers in serum for screening or monitoring before surgery, but the enzymatic method is time consuming. However, the MNP method accelerates the antigen–antibody reaction. This is a different mechanism to magnetic separation, in which antibody-immobilized MNPs can be attracted to immobilized antigen via a magnetic field. In this section, we describe two examples of the MNP method for biomarker detection: pancreas cancer-specific extracellular vesicles (EVs) using ferrite and glycidyl methacrylate (FG) beads and prostate-specific antigen (PSA) using fluorescent FG (FF) beads.

### 2.1. Measuring a Biomarker in Serum Using FG Beads

#### 2.1.1. FG Beads

**Figure 2:**
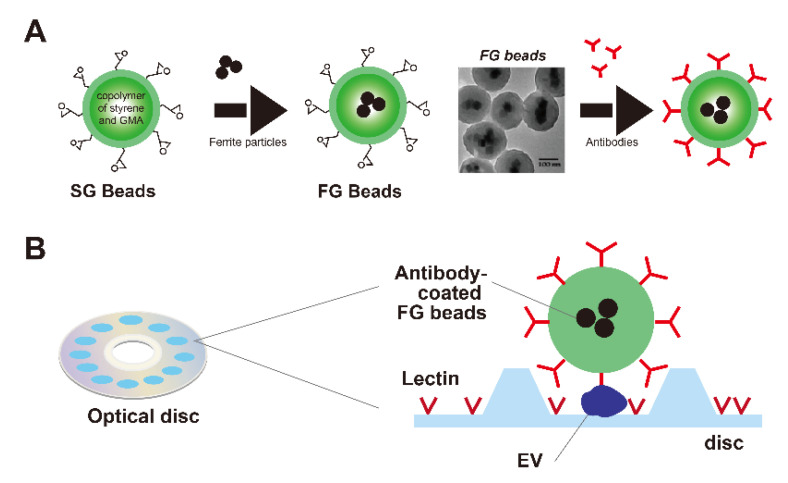
(**A**): Construction of SG and ferrite and glycidyl methacrylate (FG) beads. SG beads are composed of styrene and glycidyl methacrylate (GMA) (left). FG beads are prepared with surface-modified ferrite particles, styrene, and GMA (middle). Transmission electron microscopy image is shown (middle). Antibodies can be immobilized on the surface of FG beads (right). Modified from Inomata et al. and Nishino et al. (**B**): Schematic image of the quantification of extracellular vesicles (EVs). Candidate lectins were coated on the optical disc of the ExoCounter system. Lectin-binding EVs in the sera of pancreatic cancer patient or cell lines were captured on the disc and labeled with anti-CD9 Ab-conjugated nanoparticles. The absolute numbers of labeled EVs were quantified using the optical disc drive of the ExoCounter. Modified from Yokose et al.

Handa’s group initially developed affinity latex beads, styrene–GMA (SG) beads, which have a polystyrene core and glycidyl methacrylate (GMA) on their surface, known as poly GMA beads (Figure 2A left) [8]. Poly GMA beads have epoxy groups that can immobilize proteins, nucleic acids, and low-molecular-weight compounds. Additionally, the group found that carboxyl and thiol groups bind to the ferrite surface [9,10]. On the basis of these findings, 35–40 nm ferrite was coupled with the adaptor molecule and then coated with a copolymer of styrene and GMA, followed by coating with GMA [10] to generate the FG beads (Figure 2A, middle). FG beads have a 200 nm diameter with several encapsulated ferrite nanoparticles. Similar to the SG beads, specific ligands can be bound to the GMA surface to enable it to bind target molecules (Figure 2A, right). Because of the ferrite core, it can then be attracted or separated using magnetic forces.

#### 2.1.2. Screening or Monitoring of EVs with FG Beads

EVs are granular substances with a diameter of 50–150 nm, and they are secreted by cells [11,12]. Lipids and proteins derived from cell membranes are contained on the surface of EVs, and inside the EVs are intracellular substances, such as nucleic acids [13] (including microRNA, messenger RNA, and DNA) and proteins [14]. Recently, it has been suggested that EVs are involved in cancer development. EVs released from cancer cells are known to function in ways that favor cancer cells, such as cell survival, malignant transformation, and metastasis.

The surface proteins on EVs reflect parental cells, such as CD147 from colorectal cancer cells [15,16], human epidermal growth factor receptor 2 (HER2) from breast cancer cells [17], and CD91 from lung cancer cells [18]. Therefore, measuring specific EVs released from cancer cells has potential in cancer screening and monitoring. The methods used to count EVs are mainly conventional particle-counting methods, such as nanoparticle tracking analysis [19,20] and tunable resistive pulse sensing [21,22], or labeling-detection methods, such as ELISA [18,23] and flow cytometry [14,24].

The ExoCounter system is a unique assay system that uses FG beads to count the absolute number of EVs and analyze surface proteins simultaneously. The system uses an optical disc with periodic grooves that are 160 nm wide at the bottom and 260 nm wide at the top. Individual EVs can be bound at the bottom of the groove and FG beads at the top (Figure 2B). The basic reaction mechanism is a magnetically prompted rapid sandwich immunoassay. Using an optical head based on Blu-ray disc technology, EVs modified with nanoparticles are detected one by one. The immunoassay uses antibody-coated detection FG beads and samples placed on a capture antibody- or ligand-coated optical disc (Figure 2B). A magnet is attached under the disc for 1–2 min to concentrate the FG beads onto the immobilized capture antibody or ligand, and then unbound FG beads are washed out. The captured FG beads are counted by an optical pickup composed of a laser diode and a photodetector.

**Figure 3:**
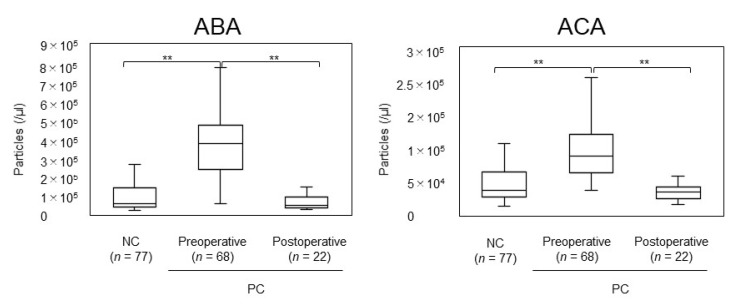
Quantification of ABA- and ACA-positive EVs from the sera of preoperative and postoperative pancreatic cancer patients and negative controls. Patient sera were analyzed using ABA- or ACA-coated discs and anti-CD9 Ab-conjugated beads with ExoCounter. Adapted from Yokose et al. ** *p* < 0.01.

The ExoCounter system has been used to analyze pancreatic cancer patient serum, in which EVs with glycoprotein are bound to Agaricus bisporus agglutinin (ABA) or Amaranthus caudatus agglutinin (ACA) using CD9 antibody-coated FG beads to detect EVs on an ABA- or ACA-coated disc [25]. Using this method, EVs that have a carbohydrate chain that binds to ABA or ACA can be detected. EV quantification was performed on 90 samples from pancreatic cancer patients (68 preoperative and 22 postoperative samples) and 77 negative control serum samples [25]. The ABA-binding and ACA-binding EVs were significantly higher in the preoperative pancreatic cancer patients than in the negative controls (*p* < 0.001 and *p* < 0.001, respectively) (Figure 3) [25]. Furthermore, the number of labeled EVs was significantly reduced in the post-pancreatectomy sera, almost to the same level as that of the negative controls (*p* < 0.001 and *p* < 0.001, respectively) (Figure 3) [25]. The measurement that captures the characteristics of EVs is quite unique.

### 2.2. Measuring a Biomarker in Serum Using FF Beads

#### 2.2.1. FF Beads

**Figure 4:**
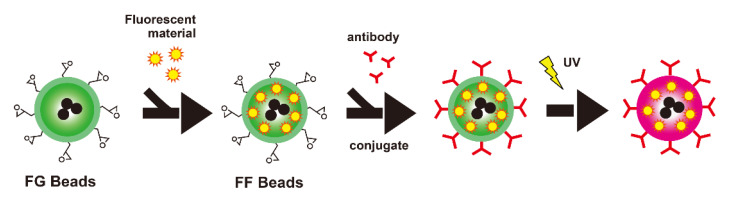
Scheme of FF beads. FF beads were prepared by encapsulating fluorescent materials in FG beads. Antibodies were immobilized on FF beads. FF beads emit fluorescence upon exposure to UV excitation. Modified from Kabe et al.

The next generation of FG beads is fluorescent FG beads (FF beads). Generally, fluorescent substances are immobilized on the polymer surface by covalency or affinity. However, a unique feature of FF beads is that fluorescent substances, such as europium complexes (Eu (TTA)_3_ (TOPO)_2_), can be encapsulated. Europium complexes emit fluorescence at 618 nm under light excitation at 340 nm. FG beads are tolerant to several organic solvents and expand or shrink depending on the type of solvent. When acetone is used, the surface polymer of FF beads swells along with the encapsulated fluorescent substance, and then returns to its original configuration in water (Figure 4). The fluorescence can be directly observed with a fluorescence detector or microscope. In addition to their magnetic attraction function, signal amplification is not necessary, which enables fast and highly sensitive disease diagnosis [26,27,28].

#### 2.2.2. Screening or Monitoring of Cancer Biomarkers with FF Beads

FF beads were used to measure PSA, a widely used biomarker in patients with prostate cancer, using a magnetically prompted rapid sandwich immunoassay [26]. Detection was undertaken by measuring the fluorescence intensity. The detected antibody-coated FF beads and samples were placed on an antibody-coated capture microplate, and a magnet was attached under the plate for 1–2 min to concentrate the FF beads onto the immobilized antibody. The unbound FF beads were washed out, and the fluorescence of the remaining FF beads was held on the plate through the antigen–antibody reaction, which was then measured directly. When the limit of quantification (LOQ) was defined as the lowest concentration measurable intraassay (CV < 20%) in the sandwich immunoassay with FF beads, then the LOQ of this method was estimated to be 0.02 ng/mL for PSA in serum [26].

Clinical examination of prostate cancer requires the detection of PSA in serum over a range of 0.1 to 10 ng/mL [29,30]. Magnetically prompted rapid sandwich immunoassay is therefore sufficient to analyze a healthy donor who would generally have low concentrations of PSA (<0.1 ng/mL) and patients with prostate cancer who would have concentrations >4.0 ng/mL [26].

## 3. Intraoperative Diagnosis during Surgery

Cancerous areas are surgically removed and diagnosed pathologically during surgery, often with lymph nodes. The powerful application of MNPs in intraoperative situations includes sentinel lymph node (SLN) mapping and the rapid diagnosis of metastasis in SLNs. Currently, radioisotope (RI) tracers and blue dye are used as the gold standard for SLN mapping during surgery [31,32]; however, the RI method risks radiation exposure to both patients and medical personnel. Furthermore, the locations at which it can be used are limited because RI methods require nuclear medicine facilities. Using biocompatible MNPs, such as SPIONs, SLN detection can be performed without a special RI facility. Moreover, this MRI contrast media can drain into SLNs faster than RI and can be detected using a magnetometer.

The resected lymph nodes can be examined pathologically during surgery. Rapid diagnosis of cancer or metastasis in SLNs is necessary for surgical decision making. To visualize cancer or metastasis, immunostaining can increase the accuracy of diagnosis, but it is usually time consuming.

In this section, magnetic methods for SLN detection and rapid immunostaining are described.

### 3.1. Detecting Sentinel Node during Surgery Using SPIONs

Lymph nodes are responsible for trapping foreign substances, such as pathogens, before they can spread throughout the body, and eliminating them through an immune response [33]. Metastasis to regional lymph nodes is the most important prognostic indicator of outcome in patients with solid tumors. Tumor cells that have invaded the stroma can reach regional lymph nodes through the lymphatic capillaries and trunks around the tumor, forming lymph node metastases [33]. In melanoma [34] and breast cancer [35], the SLN theory has been established, whereby tumor cells that invade the lymphatic vessels first metastasize to specific lymph nodes, the so-called SLNs [36], and then to regional lymph nodes and organs throughout the body.

Pathologic examination of SLNs during surgery could provide information about the staging of regional lymph nodes. If the SLN is demonstrated to be cancer negative, then radical lymph node dissection would not be necessary. Recently, the applications of SLN theory were reported to be beneficial for many cancers, such as skin [36], breast [37], gastrointestinal [38], and gynecological cancers [39]. There could even be benefits during laparoscopic surgery [40].

The standard approach for the detection of SLNs is the dual-tracer method using an RI tracer (radiolabeled tin colloid) and blue dye [41]. However, the use of RIs requires a nuclear medicine facility. Furthermore, the RI tracer must be injected 2–24 h prior to surgery for accurate SLN detection [32]. These issues indicate the need for non-radioactive, rapid-assessment tracers with an ability to reliably detect SLNs. The RI method could therefore be replaced by a magnetic method.

#### 3.1.1. SPIONs

SPIONs can be categorized as MNPs. SPIONs, such as Sienna+ and Resovist, are hydrophilic colloidal solutions of γ-Fe_2_O_3_ coated with carboxydextran. The diameter of the iron oxide particles is 4–10 nm, and the total size of SPIONs is approximately 60 nm. SPIONs are biocompatible and are specifically taken up by reticuloendothelial tissues (Kupffer cells), mainly in the liver. MRI is a diagnostic approach that uses a receiving coil to acquire the radio waves generated when a high-frequency magnetic field is applied to hydrogen atoms in a living body, causing a resonance phenomenon, and creates an image on the basis of the signal data. SPIONs are used as a negative contrast agent because they have a strong transverse relaxation time (T2) shortening effect and decrease the MR signal. After administration to the human body, SPIONs are rapidly taken up by Kupffer cells in the liver. Kupffer cells are not present in cancerous tissues and, thus, exert a contrast effect in MRI [42].

SPIONs have also been used as tracers for SLN biopsy. Following injection around the tumor, SPIONs are taken up by the SLNs and detected by a dedicated probe [43]. In this section, we focus on SLN detection by SPIONs.

#### 3.1.2. Magnetic Probes

##### Magnetic Probes for Breast Cancer

Magnetic field detectors are necessary to detect SPIONs in SLNs for SLN mapping. A number of magnetic probes have been developed. For example, Sentimag is based on the mechanism of an AC pickup coil that is commercially available and is one of the most widely used in clinical settings [43,44,45,46,47,48,49]. Other magnetic probes that are based on the fundamental mode of orthogonal fluxgate (FM-OFG) [50,51,52,53], such as a magnetic tunneling junction (MTJ) sensor [54] and negatively charged nitrogen-vacancy centers in diamonds, have been developed. DiffMag is based on a pickup coil with AC and DC differential magnetometry [55,56]. These magnetic probes have demonstrated the ability to detect between 280 ng and 500 µg SPIONs from a distance of 1 mm to 2.5 cm.

Sekino et al. [57] showed that the amount of iron uptake in SLNs in breast cancer patients was approximately 140 ± 80 µg [57], which was 0.3% of the injection dose (1.6 mL of Resovist) that contained 44.6 mg of iron. Therefore, the magnetic probe is required to have a detection ability in the order of 100 µg at a typical distance of 2–3 cm for breast cancer to be applicable in the clinic.

A magnetic probe developed by Sekino and Kusakabe’s group employed a permanent magnet and a Hall-effect magnetic sensor with a code-less handheld shape [57]. This probe is also commercially available as a medical device (Matrix Cell Research Institute Inc., Ibaraki, Japan, CE mark 93/42EEC; NB:0344, EC certificate No.4201663CE01). The major feature of this probe is that it allows precise positioning of the sensor with respect to the magnetic null point (where the magnetic flux density is zero) to remove environmental effects, such as any ambient magnetic fields and temperature effects. Other features of this probe are its easy handling for surgeons during operations because of its compact shape and low weight (108 g), and its code-less appearance. This probe can detect 56, 140, 280, and 560 µg SPIONs at a distance of 7, 9, 11, and 15 mm, respectively.

##### Magnetic Probe for Laparoscopic Study

Laparoscopic surgery is a less intensive method of surgery in which an endoscope and forceps are manipulated in four to five small incisions with ports (trocars) [58]. Usually, two sizes of ports are used, and the inner diameter of the larger port is 12 mm. Therefore, there is the need for a magnetic probe of a suitable shape for laparoscopic surgery. The differences between magnetic probes used for breast cancer and laparoscopic surgery are shown in Table 1 [57,59].

The benefit of using magnetic nanoparticles, such as SPIONs, for SLN mapping in laparoscopic surgery is not just to avoid RI exposure, but because of the speed at which SPIONs can drain to SLNs from the injection site. SPIONs drain quicker than RI tracers [60], meaning that SPIONs could be used as an SLN detection tracer during surgery. Another benefit is the detection distance, which is shorter than that of RI. Furthermore, the strength of the RI tracer signal means that signals from the injection site can interfere with the detection signal from SLNs [41,61,62]. This so-called shine-through effect is especially pronounced in the narrow intraperitoneal space and is not an issue with magnetic nanoparticles.

Kuwahata et al. [63] developed an AC/DC probe magnetic sensor for laparoscopic surgery. This probe employs a nonlinear response from the magnetic nanoparticles magnetized by an alternating magnetic field with a static magnetic field to achieve sensitive detection. The probe showed a longitudinal detection length of 10 mm for 140 µg iron; the detection limit is approximately 280 ng from a 1 mm distance. The suitability of the probe was demonstrated using a porcine model.

#### 3.1.3. SLN Detection during Surgery

##### Breast Cancer

Magnetic tracers are taken up by macrophages in the lymph nodes and detected by a handheld magnetometer [43]. In a previous study, it was shown that SPIONs reach the axillary lymph nodes within minutes after injection into the breast [60]. To detect SPIONs, several magnetometers have been developed [43,56,64].

In the EU, Sienna+ (a suspension of SPIONs) and Sentimag (a specialized probe) are used for SLN biopsy of breast cancer. Sienna+ is injected into the tumor periphery to reach the SLNs and can be identified by Sentimag. Sienna+ is a suspension of dark grains and can be recognized as a dye. A meta-analysis of clinical trials of SLN biopsies using magnetic detection systems showed that the identification rate of SLNs was not inferior to that of simultaneous administration of radiocolloid ± dye (conventional method vs. magnetic method: 96.8% vs. 97.1%).

Clinical tests using SPIONs and blue dye tracers in patients with breast cancer have shown that handheld magnetic probes are useful for detecting SLNs containing magnetic nanoparticles [65]. A multicenter study of breast cancer SLN biopsies using TAKUMI and Resovist (ferucarbotran) as a tracer showed that the identification rate of SLNs was not inferior to that of the RI method (RI method vs. magnetic method: 98.1% vs. 94.8%) [66].

##### Gallbladder Cancer

SLN mapping is challenging for cancers of difficult-to-access visceral organs, such as the gallbladder. This is because the standard method of RI use requires preoperative tracer injection. Indocyanine green (ICG) fluorescence imaging is a promising tool for SLN detection in patients with breast, gastric [67], and colorectal cancers [68]. Lymph flow and SLNs are detected soon after injection with a fluorescence imaging system, even in dense adipose tissue. However, because the ICG tracer is small, it passes through downstream lymph nodes, making it difficult to quantitatively analyze SLNs [69]. Magnetic methods to detect intra-abdominal SLNs can be used to overcome these challenges and have been effectively applied.

**Figure 5:**
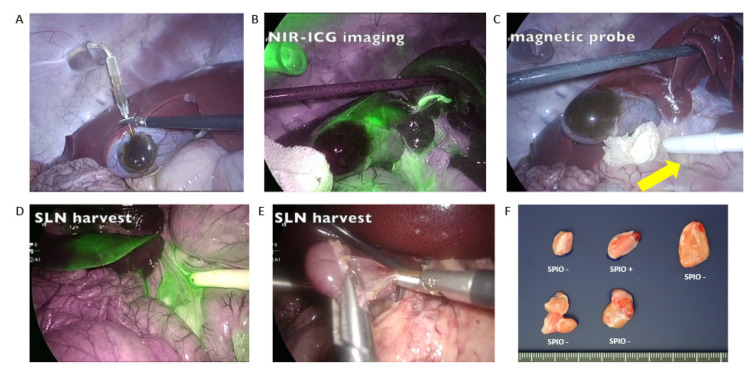
Laparoscopic sentinel lymph node (SLN) detection with a mixed tracer in porcine surgery. (**A**): Injection of the mixed indocyanine green (ICG) dye and magnetic tracer into the gallbladder wall. (**B**): ICG fluorescence signals detected by near-infrared laparoscopy. (**C**): Magnetic field evaluation of lymph nodes with the laparoscopic magnetic probe (yellow arrow). (**D**): Fluorescence signal-oriented identification of SLNs by the magnetic method. (**E**): Resection of the detected SLNs. (**F**): Brown pigmentation with the magnetic tracer in one resected regional lymph node among five. Modified from Mihara et al.

In a gallbladder cancer feasibility study using an animal model, the TAKUMI probe, which includes a Hall sensor, was modified for laparoscopic use [59]. Its feasibility for detecting SLNs of the gallbladder was evaluated using a laparoscopic dual-tracer method by injecting ICG and SPIONs into five wild-type pigs without cancer and one immunodeficient (RAG2-knockout) cancer-bearing pig. The laparoscopic probe identified the SPIONs in the lymph nodes of four out of the five wild-type pigs during surgery (Figure 5). The magnetic field counts were 2.5–15.9 µT, and fluorescence was detected in SLNs in all five pigs.

ICG shows a visual lymph-flow map, and SPIONs more accurately identify each SLN with a measurable magnetic field, which is similar to the RI method. It was confirmed using a RAG2-knockout porcine gallbladder cancer model with lymph node metastases that SLN mapping is effective under tumor-burden circumstances. We identified an SLN in the laparoscopic investigation, and the magnetic field count was 3.5 µT. The SLN was histologically determined to be one of two metastatic lymph nodes [59]. This result suggested the possibility of identifying SLNs in the intra-abdominal cavity organs.

### 3.2. Magnetically Promoted Rapid Immunofluorescence (MRIF) Staining Using FF Beads

Resected SLNs are examined pathologically. Here, we describe the rapid immunostaining of SLNs with positive images observed by fluorescence microscopy.

#### 3.2.1. Europium Single Staining

**Figure 6:**
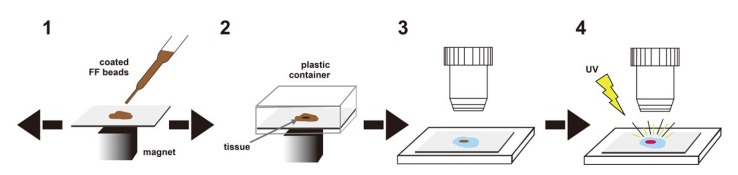
Scheme of magnetically promoted rapid immunofluorescence. 1: Diluted FF beads are dripped onto tumor cells, and the slide is vigorously agitated on the magnet; 2: the slide is inverted into a plastic container and washed with a magnet; 3, 4: FF beads bound to tumor cells can be observed directly by fluorescence microscopy. Modified from Onishi et al.

**Figure 7:**
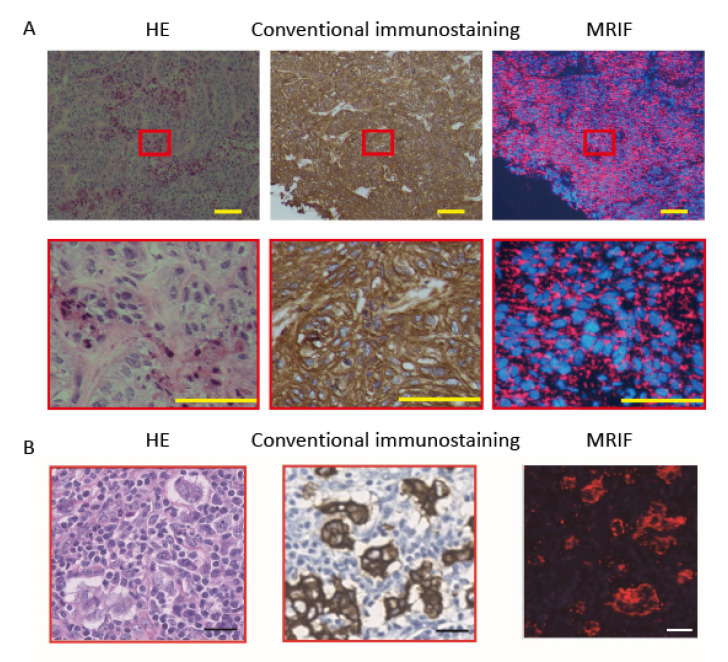
(**A**): Staining of A431 cells by hematoxylin–eosin (HE) (**left**), conventional immunostaining (**middle**), and MRIF (**right**). Images of an A431 (human epidermoid cancer cells with high expression of epidermal growth factor receptor (EGFR)) xenograft in pigs. (**B**): Staining image of a human breast cancer metastatic lymph node by HE (**left**), conventional immunostaining (**middle**), and MRIF (**right**) incubated with anti-pan-cytokeratin antibody-coated FF beads. Image of a paraffin-embedded tissue array of a stained human breast cancer metastatic lymph node. Scale bar = 250 µm and 25 µm for high magnification. Adapted from Onishi et al.

Accurate identification of the extent of a lesion allows the surgeon to minimize removal during minimally invasive surgery of solid tumors. Thus, there is a need for the rapid diagnosis of lesion characteristics and progression during surgery [70,71]. Generally, snap-frozen sections are prepared during surgery and stained with hematoxylin–eosin (HE) for examination by a pathologist. Although HE staining can provide rapid diagnosis, diagnosis can be difficult, such as in cases with small lesions. Immunostaining is one approach to increase the diagnostic accuracy. The avidin–biotin complex method is a commonly used immunostaining system that involves four sequential steps: (1) primary antibody staining; (2) biotin-labeled secondary antibody staining; (3) avidin–biotin–peroxidase complex formation; and (4) development by diaminobenzidine (DAB) staining. Antigen–antibody reaction steps by primary and secondary antibodies are particularly time consuming, and the method is not suitable for rapid intraoperative diagnosis. Thus, there have been attempts to shorten the time of the procedure using ultrasound [72] and microwaves [72,73] that accelerate the antigen–antibody reaction with a stirring effect in addition to Brownian motion. Alternatively, Onishi et al. used FF beads to develop MRIF staining, which shortens reaction and washing times using a magnet [26,74]. MRIF can be performed in two steps without secondary antibody, signal amplification, or DAB staining: (1) incubation with antibody-coated FF beads and (2) washing, because the antigen–antibody complex can be directly observed using a fluorescence microscope to observe the fluorescent material encapsulated in the FF beads (Figure 6). This procedure reduces the time to a 1 min reaction and 1 min wash step with a magnet when applied to frozen sections of xenografted samples of A431 human epidermoid cancer cells that express high levels of epidermal growth factor receptor (EGFR) and anti-EGFR antibody-europium encapsulated FF beads (Figure 7A) [74].

The strength of the magnetic force is critical for obtaining maximum results; therefore, a jig was prepared, and the relationship between the magnetic force and the distance from a 10 mm diameter and 24 mm length cylindrical magnet was examined. The magnetic force (F) acting on an FF bead was calculated as F = −∇(−*m_b_*⋅*B*), where *m_b_* is the magnetic moment of the FF beads, and B is the magnetic field strength of the magnet [75]. The distribution of the magnetic force was stronger at the margins than at the center of the magnet; therefore, we decided to agitate the magnet to obtain uniform staining [74]. The optimal distance between the A431 xenograft samples and the magnet using anti-EGFR antibody-coated FF beads for a 1 min incubation was within 2–5 mm, whereby the magnetic force =7.79 × 10^−15^ N to 3.35 × 10^−15^ N. A distance shorter than 2.0 mm showed unwanted background staining, and a distance greater than 5 mm showed insufficient staining. We also examined the optimal distance for washing. A distance from the samples to the magnet of 11 mm with a magnetic force of 4.78 × 10^−16^ N showed the best result for anti-EGFR antibody-coated FF beads. A distance >11 mm showed unwanted background staining. The staining efficiency was confirmed by the staining of breast cancer clinical samples for cytokeratin (CK), which is present in all epithelial cells, even in tumorigenesis, and is a widely used epithelial marker. Anti-pan-cytokeratin antibody (AE1/AE3) was used in this study. Figure 7B shows similar patterns of staining by conventional immunostaining and MRIF, which is consistent with the cancer region observed in the HE-stained section. The positive rates of conventional immunostaining were compared with MRIF staining using anti-pan-cytokeratin antibody-coated FF beads and clinical tissue array samples. The positive rate of conventional immunostaining was 96.5% (276/286) and that of MRIF was 92.7% (265/286). The coincidence rate was 94.8% (271/286) [74]. Normal tissue (i.e., breast tissue, tonsil, and lymph nodes) was analyzed. The positive rate of conventional immunostaining was 26.3% (25/95) and that of MRIF was 32.6% (31/95) [74]. The coincidence rate was 91.6% (87/95) (Table 2). Under optimal conditions, this ultrarapid immunostaining approach may be an ancillary method for pathological diagnosis during surgery.

#### 3.2.2. Multi-Colored Staining

**Figure 8:**
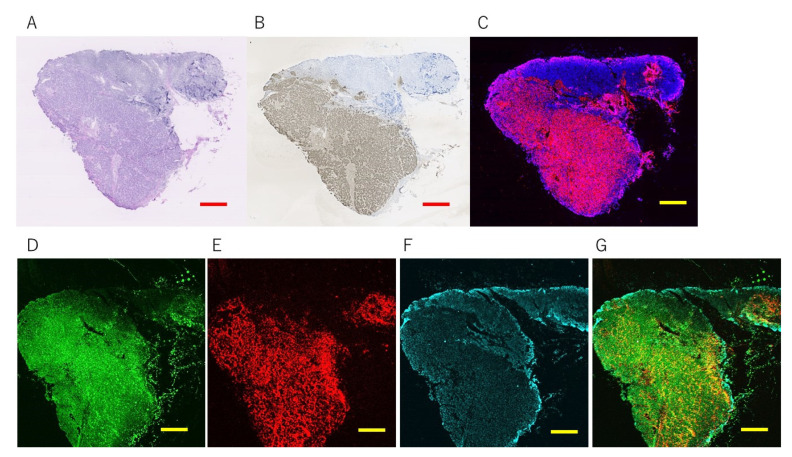
Staining of a frozen tissue section of a human breast cancer metastatic lymph node. (**A**): Hematoxylin and eosin (HE) staining. (**B**): Conventional immunostaining (diaminobenzidine). (**C**): Single staining of magnetically promoted rapid immunofluorescence (MRIF) with anti-pan-cytokeratin antibody-coated FF beads that emitted magenta fluorescence. Multi-colored MRIF using anti-CK19 antibody-coated F beads that emitted green fluorescence (**D**), anti-TNC antibody-coated FF beads that emitted red fluorescence (**E**), DAPI staining (**F**), and merged images (**G**). Scale bar = 1000 μm Adapted from Onishi et al. and new data.

Using several hydrophobic fluorophores that can be embedded into the polymer layers of the beads, the construction of multi-colored FF beads becomes possible. We applied a series of compounds, such as 3-dimesityl boryl-2,2′-bithiophene and 5,5′-dimesityl-3-dimesityl boryl-2,2′-bithiophene, which contain boron, to create multi-colored FF beads (patent: JP 6409173). Through the fluorescent labeling of target markers, multi-MRIF would be achieved. Figure 8 shows HE staining, conventional IHC staining, europium single staining, and europium double staining of human lymph nodes with metastasis by multi-MRIF. We designed antibody-coated FF beads to emit fluorescence independently. FF beads were coated with antibodies against CK19, which is expressed in epithelial cells, and tenascin C (TNC), which is a glycoprotein that is expressed in the extracellular matrix around cancer cells. Because some triple-negative breast cancers do not express CK19, tenascin C is a good candidate to compensate for CK19 to increase the detection rate of triple-negative breast cancer. Anti-CK19 antibody-coated FF beads show green fluorescence, and anti-TNC antibody-coated FF beads show red fluorescence. Conventional immunostaining with pan-CK was well correlated with single MRIF staining with pan-CK antibody-coated FF beads, which showed magenta fluorescence derived from europium complexes. The blue color was nuclear with DAPI staining. For CK19 and TNC double staining, both sets of FF beads were equally mixed and stained under the same magnetic conditions as EGFR for a 1 min reaction and a 1 min wash. CK19 (Figure 8D) and tenascin C (Figure 8E) were mostly stained in cancerous regions. Figure 8G shows merged images from D, E, and F. There is still a need to optimize the conditions because the antibody affinity is varied; however, this result demonstrates the possibility of double staining in one step. Furthermore, when frozen sections of six human metastatic lymph node samples from breast cancer were stained with IHC and MRIF, all lymph nodes were positive with a 100% concordance rate. In short, we successfully performed fluorescence multiplex staining of human breast cancer metastatic lymph nodes by binding antibodies against CK19 and TNC to FF beads containing different fluorophores. Because the system is applicable to frozen sections, it enables rapid diagnosis and meets clinical needs.

## 4. Discussion and Future Perspectives

In this review, we described the applications of MNPs in oncology from a surgeon’s perspective of monitoring biomarkers before and after surgery, and for intraoperative diagnosis during surgery. Pancreatic cancer-specific EVs and a cancer-specific antigen, PSA, were measured by the magnetic method, which could be used for monitoring cancer development before and after surgery. SLN detection can be performed during surgery by the magnetic method, and immunostaining can even be completed during surgery. Laboratory techniques related to surgical procedures can be undertaken magnetically.

Notably, EV detection and immunostaining are quite unique. Most EV methods require an extraction step of EVs from serum or plasma, but this magnetic method uses serum directly and involves counting the absolute number of EVs that express characteristic cell surface proteins. Pancreatic cancer is one of the most aggressive cancer types because it is difficult to identify in the early stages [76]. This method has potential as an early detection tool. Immunostaining is a powerful tool to increase the accuracy of diagnosis, but to contribute to decisions on surgical procedure, staining must be completed within 20 min [77]. MRIF requires only a 1 min reaction and a 1 min wash, and, thus, this method has the potential for practical application in the clinic. Moreover, because it is easy to define MRIF as positive and negative, it can be automated, reducing the requirement for a pathologist.

The magnetic SLN method is a promising alternative to the RI method. Moreover, it has the potential for clinical application to the laparoscopic method for detecting SLN metastasis from cancers of visceral organs, which are difficult to examine via the surface of the body or by endoscopy. These procedures can enable the identification of SLNs for almost all intra-abdominal organs that are laparoscopically accessible. Moreover, the long shelf life and easy handling of SPIONs and their detector permit the accurate diagnosis of metastatic cancers in mid- to small-scale medical facilities and developing countries.

Because europium is toxic, FG beads also have the potential for magnetic sensing with magnetic probes. Magnetic sensing activities strongly depend on magnetic characteristics, such as the magnetic moment. Compared with the magnetic moment of Resovist (approximately 50 emu/g) [78], the magnetic moment of the beads (20 emu/g) [10] is relatively small. Considering the detectable distance of Resovist of 9 mm with a magnetic probe as demonstrated by Sekino et al. [57], the detectable distance of the FF beads could be several millimeters. This expected magnetic sensing activity potentially enables the intra-abdominal detection of cancer and lymph nodes at a proximal distance.

The problems relating to the rate of false positives and false negatives that this type of methodology generates in each of its applications should be addressed, for example, which test confirms that the biological matrix has correctly come into contact with the analytical system in the presence of a negative result. However, regarding EV measurement, a lectin array [25] could be used to confirm the result; however, the results of lectin arrays are relative and are not quantitative. PSA measurements should be confirmed by conventional methods, such as ELISA, but the authors did not examine the associated rate of false positives and false negatives. The sentinel node is defined as the first lymph node that cancer cells reach, and the number of nodes may vary depending on the detection method. There are usually one or two for the RI method and more for the dye method. It is therefore difficult to discuss false positives and false negatives. In this review, we described the ICG dye method and the SPION method. Regarding MRIF staining, Onishi et al. [74] used conventional immunostaining to confirm that the antibody had correctly come into contact with the antigen and described the concordance rate because tissue array samples are not always serial sections.

## 5. Conclusions

Screening, monitoring, and diagnosis are critical in oncology treatment. However, current clinical methods are time consuming. The use of magnetic nanoparticles is an alternative approach that offers faster analyses over a wide range of oncological applications, such as the detection of cancer biomarkers and immunostaining. Radioisotope tracers are used for SLN mapping during cancer surgery; however, the RI method risks radiation exposure to both patients and medical personnel and requires nuclear medicine facilities. Using biocompatible MNPs, such as SPIONs, SLN detection can be performed safely without a special RI facility. The magnetic method is an interesting approach and its use is expected in more applications. It is hoped that large-scale clinical trials will be undertaken to demonstrate its usefulness and to validate it for clinical diagnosis.

## Figures and Tables

**Table 1:** Comparison of the probes for breast cancer and laparoscopic surgery.

Measure	Probe for Breast Cancer	Probe for Laparoscopic Surgery
Appearance	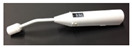	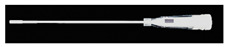
Total length	24.5 cm	58.5 cm
Handle length	16 cm	17 cm
Shaft length	7 cm	37 cm
Head size (diameter)	18 mm	10 mm
Weight	100 g	150 g
Detection range (140 μg Resovist)	8 mm	6mm

**Table 2:** Coincidence ratio between conventional IHC and MRIF staining.

Immunostaining Method	MRIF			
	Result	+	−	Total
**Conventional Immunostaining**	**+**	263	13	276
	**−**	2	8	10
	**Total**	265	21	286

MRIF, magnetically promoted rapid immunofluorescence.
